# Fucoidan-derived carbon dots against *Enterococcus faecalis* biofilm and infected dentinal tubules for the treatment of persistent endodontic infections

**DOI:** 10.1186/s12951-022-01501-x

**Published:** 2022-07-14

**Authors:** Shang Tang, Hui Zhang, Li Mei, Keke Dou, Yuying Jiang, Zhanyi Sun, Shuai Wang, Mohamed Sayed Hasanin, Jing Deng, Qihui Zhou

**Affiliations:** 1 Department of Stomatology, The Affiliated Hospital of Qingdao University, Qingdao, 266003 China; 2 School of Stomatology, Qingdao University, Qingdao, 266003 China; 3 Institute for Translational Medicine, The Affiliated Hospital of Qingdao University, Qingdao University, Qingdao, 266021 China; 4 State Key Laboratory of Bioactive Seaweed Substances, Qingdao Bright Moon Seaweed Group Co., Ltd., Qingdao, 266400 China; 5 Cellulose and Paper Department, National Research Centre, Dokki, 12622 Cairo Egypt; 6 University of Health and Rehabilitation Sciences, Qingdao, 266071 China; 7 Dental Digital Medicine & 3D Printing Engineering Laboratory of Qingdao, Qingdao, 266003 China

**Keywords:** Fucoidan, Carbon dots, Reactive oxygen species, *Enterococcus faecalis*, Biofilms, Endodontic infection

## Abstract

*Enterococcus faecalis* (*E. faecalis*) biofilm-associated persistent endodontic infections (PEIs) are one of the most common tooth lesions, causing chronic periapical periodontitis, root resorption, and even tooth loss. Clinical root canal disinfectants have the risk of damaging soft tissues (e.g., mucosa and tongue) and teeth in the oral cavity, unsatisfactory to the therapy of PEIs. Nanomaterials with remarkable antibacterial properties and good biocompatibility have been developed as a promising strategy for removing pathogenic bacteria and related biofilm. Herein, carbon dots (CDs) derived from fucoidan (FD) are prepared through a one-pot hydrothermal method for the treatment of PEIs. The prepared FDCDs (7.15 nm) with sulfate groups and fluorescence property are well dispersed and stable in water. Further, it is found that in vitro FDCDs display excellent inhibiting effects on *E. faecalis* and its biofilm by inducing the formation of intracellular and extracellular reactive oxygen species and altering bacterial permeability. Importantly, the FDCDs penetrated the root canals and dentinal tubules, removing located *E. faecalis* biofilm. Moreover, the cellular assays show that the developed FDCDs have satisfactory cytocompatibility and promote macrophage recruitment. Thus, the developed FDCDs hold great potential for the management of PEIs.

**Graphical Abstract:**

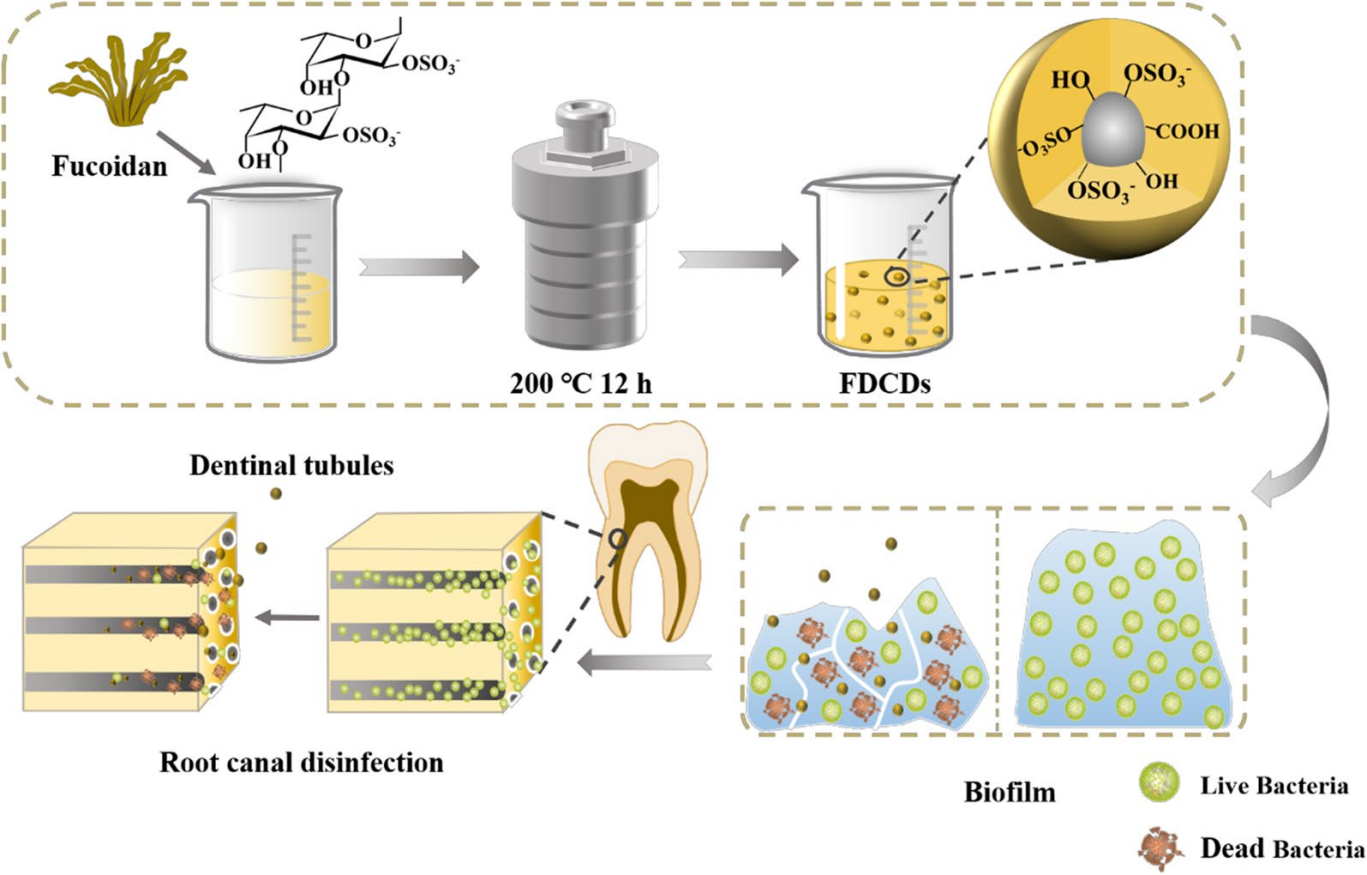

**Supplementary Information:**

The online version contains supplementary material available at 10.1186/s12951-022-01501-x.

## Introduction

Persistent endodontic infections (PEIs) are one of the most frequent diseases in the oral cavity, resulting in gum fistula, alveolar abscess, chronic periapical periodontitis, root resorption, and even tooth loss [1]. Because a variety of bacterial species were presented in the oral cavity, microorganisms and their by-products are associated with polymicrobial EIs [2, 3]. Particularly, *Enterococcus faecalis* (*E. faecalis*) related biofilms are covered in the root canals and invade into dentinal tubules, which may result in persistent infection and refractory periapical periodontitis [4, 5]. Meanwhile, because of the complex root canal system (e.g., dentinal tubules, isthmuses, the apical delta, etc.), the biofilms in these areas are difficult to remove [6]. Root canal disinfectants were widely used to eliminate the microbes in root canals [7]. However, clinical root canal disinfectants, such as chlorhexidine acetate, cresol and formaldehyde solution, calcium hydroxide, possess inherent defects that bring risks to patients and affect their long-term applications [8]. Nowadays, nanomaterials have been widely used in EIs to inhibit bacteria in the root canals [9].

In this decade, carbon-based nanomaterials have been used as antibacterial materials owing to their excellent physicochemical properties and good biocompatibility [10–12]. Particularly, carbon dots (CDs), a new member of the carbon-based material family, have gained growing attention since they were accidentally discovered in 2004 [13–15]. CDs with small size, water-solubility, photoluminescence property, low toxicity, and high biocompatibility, possess a wide range of applications in bioimaging, biosensors, detection probes, and drug delivery [16–19]. CDs have also shown outstanding antibacterial and antibiofilm effects in recent years to broaden their applications [20–22]. It was demonstrated that the synthesized CDs with extremely small size can retain the functional groups of organic precursors and related (bio)physicochemical characteristics, which enhanced their antibacterial effect [17, 23–25]. Among many organics, fucoidan (FD) derived from brown algae is a sulfated polysaccharide with multiple biological activities, such as antibacterial, antiviral, anti-tumor, anticoagulant, and immunomodulatory activities [26–32]. It was previously found that FD at high concentrations had antibacterial activities on opportunistic pathogenic bacteria (e.g., *Escherichia coli* and *Vibrio cholerae*) and oral pathogenic bacteria (e.g., *Streptococcus mutans* and *Helicobacter pylori*), which were related to the presence and content of sulfate radicals (SO_4_^2−^) [33–35]. Also, FD could recruit and activate the macrophages, which is conducive to further removing the invading bacteria [36–38]. Therefore, FD-derived CDs (FDCDs) would be an excellent candidate as a root canal disinfectant for the treatment of PEIs, which has not been reported.

In this work, FDCDs were developed *via* a facile and green strategy to investigate their effects on the treatment of PEIs. The physicochemical properties of the FDCDs were measured by different measurements. The antibacterial/biofilm test and underlying mechanisms of FDCDs were performed in vitro. Furthermore, the antibacterial properties of FDCDs on the *E. faecalis* in the root canals and infected dentinal tubules were studied. Finally, the cytocompatibility of FDCDs and their effect on macrophages was also detected. Thus, our work indicated that the developed FDCDs hold great potential for the management of persistent endodontic infections.

## Materials and methods

### Materials

FD with a molecular weight (Mw) of 276 kDa, sulfate content of 29.65%, and purity of 95% was provided from Qingdao Bright Moon Seaweed Group Co., Ltd. (China). Brian Heart Infusion (BHI), agar, tetramethylrhodamine isothiocyanate (TRITC)-phalloidin, and 4′6-diamidino-2-phenylindole (DAPI) solution were purchased from Solarbio Science & Technology Co., Ltd. (Beijing, China). The 1,3-diphenylisobenzofuran (DPBF) and 2,7-dichlorodihyrofluorescein diacetate dye (DCFH-DA) was provided from Aladdin Bio-Chem Technology Co., Ltd (Shanghai, China). The LIVE/DEAD™ BacLight™ bacterial viability kit was obtained from Thermo Fisher Scientific (UK). Cell Counting Kit-8 (CCK-8) kit was purchased by Vazyme Co., Ltd. (Nanjing, China). All reagents were used directly without further purification. Deionized (DI) water was used in the experiment.

### Synthesis of FDCDs

FDCDs were synthesized through a common hydrothermal method. Briefly, 0.8 g FD was dissolved in 40 mL DI water and stirred for 20 min at room temperature. The 2% FD solution was transferred to a 50 mL Teflon-lined autoclave and treated for 12 h at 200 °C. The dark brown solution was cooled down and centrifuged at a speed of 10,000 rpm/min for 15 min to remove the insoluble residue. The solution was dried in a vacuum oven at 80 ℃.

### Characterization of FDCDs

The morphology and size of FDCDs were detected by transmission electron microscopy (TEM, Mic JEM-1200EX, Japan). The FDCDs were diluted with DI water, ultrasonically dispersed for the appropriate time, and then observed by TEM. Then, 100 particles were randomly selected to measure their diameter with Image J software.

The fluorescence phenomenon of FD and FDCDs was recorded under natural light as well as 365 nm UV light. The PL excitation and emission spectra of the prepared FDCDs at the concentration of 0.5 mg/mL were measured using a microplate reader (Bio Tek, Synergy TM H1/H1M, USA).

The functional groups of FD and FDCDs were analyzed using the Fourier transform-infrared (FT-IR) spectrometer (Thermo Fisher Scientific, Waltham, MA, USA) around the range of 4000–500 cm^−1^. X-ray photoelectron spectroscopy (XPS) of FDCDs was performed based on PHI5000 Versaprobe III ultrahigh vacuum (1 × 10^− 9^ bar) with an Al Kα X-ray source and a monochromator.

The FD and FDCDs were dispersed in DI water at the same concentration. The zeta potentials of these samples were determined by a Zetasizer Nano ZSE (UK) which performed dynamic light scattering at 25 °C. Also, the particle size and polydispersity index (PDI) of FDCDs were measured with dynamic light scattering (DLS).

### Antibacterial assay

The frozen *E. faecalis* ATCC 29212 was activated and quadrant-streaked on a BHI agar plate. A single *E. faecalis* colony from the BHI agar plate was cultured in 8 mL of BHI with overnight shaking at 37 °C. The bacterial suspension was diluted with BHI media and subsequently obtained the fixed OD600 value at 0.03−0.05.

#### Anti-*E. faecalis* test

FDCDs with final concentrations of 1, 2, 3, 4 mg/mL were co-cultured with bacterial suspensions. BHI medium co-cultured with bacterial was a negative control group. In order to compare the antibacterial properties of FD and FDCDs, FD was also co-cultured with bacteria as the control group. All of the above groups were divided into light and dark groups. The culture tubes in the light group were exposed to visible light using a biochemical incubator (LRH-70 F, Yiheng, China). After the incubation for 12 h at 37 °C, the suspensions of each sample were diluted to a suitable concentration, dropped onto BHI agar plates with a volume of 20 µL, evenly distributed, and cultured at 37 °C for 24 h. The experiment was repeated thrice for each group. The inhibition ratio of FDCDs was assessed according to the following equation:

$${\text{Inhibition}}\;{\text{Ratio}}\;\left( \% \right) = \frac{{{\text{CFU}}\;\left( - \right)\; - \;{\text{CFU}}\;\left( {\text{s}} \right)}}{{{\text{CFU}}\;\left( - \right)}}\; \times \;100\%$$

where CFU (−) and CFU (s) are the number of bacteria per milliliter as the negative control and experimental samples, respectively.

#### Morphological changes of *E. faecalis*

The changes of the bacterial morphology and the integrities of the bacterial membrane were observed by TEM and SEM. In brief, the blank, FD, and FDCDs (3 mg/mL) groups were co-cultured with bacteria suspensions for 12 h. Then they were centrifuged at 5000 rpm for 5 min, washed three times with PBS, and fixed with 2.5% (v/v) glutaraldehyde. 200 µL of bacterial suspension was taken by TEM, and the remaining bacterial suspension was dehydrated with an ethanol gradient and examined by SEM.

#### Evaluation of extracellular/intracellular ROS formation

To detect the production of ROS, DPBF as the singlet oxygen (^1^O_2_) probe was used [39]. The absorption decrease at 410 nm was due to the decomposition of DPBF caused by ROS. Firstly, DPBF was dissolved in acetonitrile to obtain a yellow solution. FDCDs powder was dispersed in absolute ethanol (1 mg/mL). The DPBF solution was added to FDCDs ethanol solution, and the above solution was divided into two equal parts. One part was completely protected from light, and the other part was irradiated with visible light (660 nm, 3.2 mW/cm^2^) for 30 min. Then, the absorption peak at 410 nm was measured every 5 min *via* UV–vis spectrometer. The DPBF in acetonitrile under visible light and dark conditions was chosen as the control. Based on the above experiment, it has been proved that FDCDs possessed fluorescence at 410 nm. To exclude the influence of FDCDs, the FDCDs ethanol solution without DPBF was also selected as the control group. To detect the production of ROS inside the bacteria, DCFH-DA as an intracellular ROS probe was used. After being co-cultured for 12 h, the suspensions of each group were thoroughly mixed and divided into two parts equally. One part was used to count the number of bacteria by CFU count test, and another part was used to detect the ROS. The supernatant was removed after the suspensions were centrifuged (5000 rpm, 5 min). The *E. faecalis* at the bottom of the test tubes were mixed with DCFH-DA solution which was prepared according to the instructions. Under the conditions of excitation wavelength of 488 nm and emission wavelength of 525 nm, the fluorescence intensity was measured with a microplate reader (Bio Tek, Synergy TM H1/H1M, USA). Finally, the fluorescence intensity in each bacterium was estimated. In addition, the ^1^O_2_ was further detected by electron spin resonance spectroscopy (ESR) at 365 nm UV light and dark for 5 min.

### Biofilm inhibition measurement

After being sterilized with 75% alcohol, the glass coverslips with the 14 mm diameter were put into a 24-well plate. 500 µL *E. faecalis* suspension (OD600 = 0.05) was dripped into the 24-well plate and incubated in a constant environment for 48 h to form biofilms for subsequent experiments. BHI (negative control), 3 mg/mL FDCDs, 1% NaClO (positive control) groups were co-cultured with *E. faecalis* biofilms for 12 h. Using PBS to remove excess bacteria and particles, biofilms were stained by the LIVE/DEAD™ BacLight™ Bacterial Viability Kit for 20 min. After washing with PBS, a confocal laser scanning microscope (CLSM, Leica TCS SP8, Germany) was used to visualize the fluorescence imaging of the biofilms.

### Antibacterial assay in dentin blocks

#### Preparation of tooth specimens

18 mature human premolars without caries and periodontitis for orthodontic reasons were collected. After pulped tooth specimens, root canals were dredged with K-files and shaped to size F3 with ProTaper. After the use of each instrument, root canals were irrigated with 1% NaClO using EndoUltra Ultrasonic. Apical foramina of tooth specimens were sealed with fluid resin (3 M, USA). Teeth with complete root canal preparation were immersed in 3% NaClO for 24 h for disinfection and autoclaved at 121 ℃ for 30 min.

#### Infection of tooth specimens

After the sterilization, each tooth was separately placed in a 5 mL sterile tube. The 1 mL *E. faecalis* suspension which was cultured overnight in BHI medium was pipetted to every root canal. The same volume of BHI medium (3 mL) was added to each sterile tube. All specimens were incubated at 37 ℃ for 21 days to permit the formation of biofilm in the dentinal tubules. The medium was refreshed every 2 days.

#### Anti-*E. Faecalis* in dentin blocks

The 18 infected teeth specimens were randomly divided into three groups: (1) PBS (negative control); (2) 3 mg/mL FDCDs solution; (3) 1% NaClO (positive control). One mL samples were dripped into the root canal of each specimen and incubated for 12 h. Three samples from each group were chosen for scanning electron microscope (SEM). After being fixed with 2.5% (v/v) glutaraldehyde overnight, tooth specimens that were split along the long axis were sequentially dehydrated using a variety of alcohol concentrations (30%, 50%, 70%, 80% 90%, and 100%). SEM (VEGA3, TESCAN, Czech) was used to observe the distribution and morphology of *E. faecalis* in the root canals and dentinal tubules.

### Cell assays

#### Cell culture

To explore the biocompatibility of the FDCDs, mouse embryo-osteoblast precursor (MC3T3-E1) cells obtained from the Cell Center of Shanghai Institutes for Biological Sciences were selected for subsequent experiments. MC3T3-E1 cells were resuscitated and cultured in Dulbecco’s modified eagle medium (DMEM) augmented with 10% fetal bovine serum (FBS) and 1% penicillin/streptomycin in 5% CO_2_ at 37 ℃.

#### Cytotoxicity assay

The viability of MC3T3-E1 cells was evaluated by a CCK-8 assay. The cell activity of different samples was calculated by the absorbance values of FDCDs with DMEM (negative control) and 1% NaClO (positive control). MC3T3-E1 cells were transferred to 96-well plates at a concentration of 5 × 10^3^ cells/well and incubated for 24 h in a CO_2_ incubator at 37 °C. Briefly, FDCDs were dissolved in DMEM medium to acquire various concentrations of FDCDs (1, 2, 3, and 4 mg/mL). 200 µL FDCDs with different concentrations were co-cultured with cells in each well for 12, 24, and 36 h. DMEM and 1% NaClO were used as negative and positive controls. After washing with PBS, 100 µL fresh DMEM with 10 µL of CCK-8 reagent solution was poured into each well and the solutions were incubated for 1 h in the dark. Subsequently, the absorbance at 450 nm was obtained using a microplate reader (SynergyH1/H1M, Bio-Tek, China). The cell viability was assessed according to the following equation:

$$\text{Cell viability} ({\%})=\frac{\text{OD450(s)}}{\text{OD450(-)}} \times 100{\%}$$

where OD450 (s) and OD450 (−) are the absorbances values at 450 nm as experimental samples and the negative control, respectively.

Cell morphology was detected by the fluorescence staining. 5 × 10^3^ cells were added to 96-well plates and cultured at 37 °C under 5% CO_2_ for 24 h. After being co-cultured with different concentrations of FDCDs, the cells were fixed with 4% paraformaldehyde (PFA) for 20 min and treated with 0.5% Triton X-100 for 3 min. Finally, the cells were stained with TRITC-Phalloidin and DAPI for the cytoskeleton and nucleus according to the manufacturer’s instructions. The different images of the cells were captured via inverted fluorescence microscopy (Nikon A1 MP, Japan). Image J software was used to measure the length and width of 50 cells in each sample. The elongation was calculated by the following equation:

$${\text{Elongation}}=\frac{{\text{length}}}{{\text{width}}}$$

#### Macrophage morphology and migration

RAW 264.7 cells were resuscitated and cultured in DMEM augmented with 10% FBS in 5% CO_2_ at 37 ℃. The migratory property of RAW 264.7 cells was detected using a conventional 24-well Transwell system (8 μm pore size, Corning, NY, USA). 500 µL of FDCDs which was dissolved in DMEM (3 mg/mL) was added to the lower well and 5 × 10^5^ macrophages were seeded in each upper chamber. The DMEM in the lower chamber was used as the negative control. The 24-well was incubated for 12 and 48 h. Forward, the upper chamber was washed with PBS slightly and fixed in 4% PFA for 30 min. After fixation, the drying chamber was stained with 0.1% crystal violet in the dark for 15 min. Next, the chamber was washed with DI water, and the adherent cells were photographed by a microscope. Then quantitative calculation by the following formula:

$${\text{Migration}}\;{\text{Index}}\left( \% \right) = \frac{{{\text{Count}}({\text{s}})}}{{{\text{Count}}( - )}} \times 100\%$$

where Count (s) and Count (−) are the number of macrophages after co-cultured with FDCDs and DMEM, respectively.

### Statistical analysis

All data points were presented as mean values ± standard deviation (mean ± SD). Statistical analysis was calculated using GraphPad Prism 8.0. One-way analysis of variance (ANOVA) with Tukey’s test was used to analyze differences between groups. Different numbers of asterisks indicate the significance differences, *p < 0.05, **p < 0.01, and ***p < 0.001.

## Result and discussion

### Synthesis and characterization of FDCDs

**Fig. 1:**
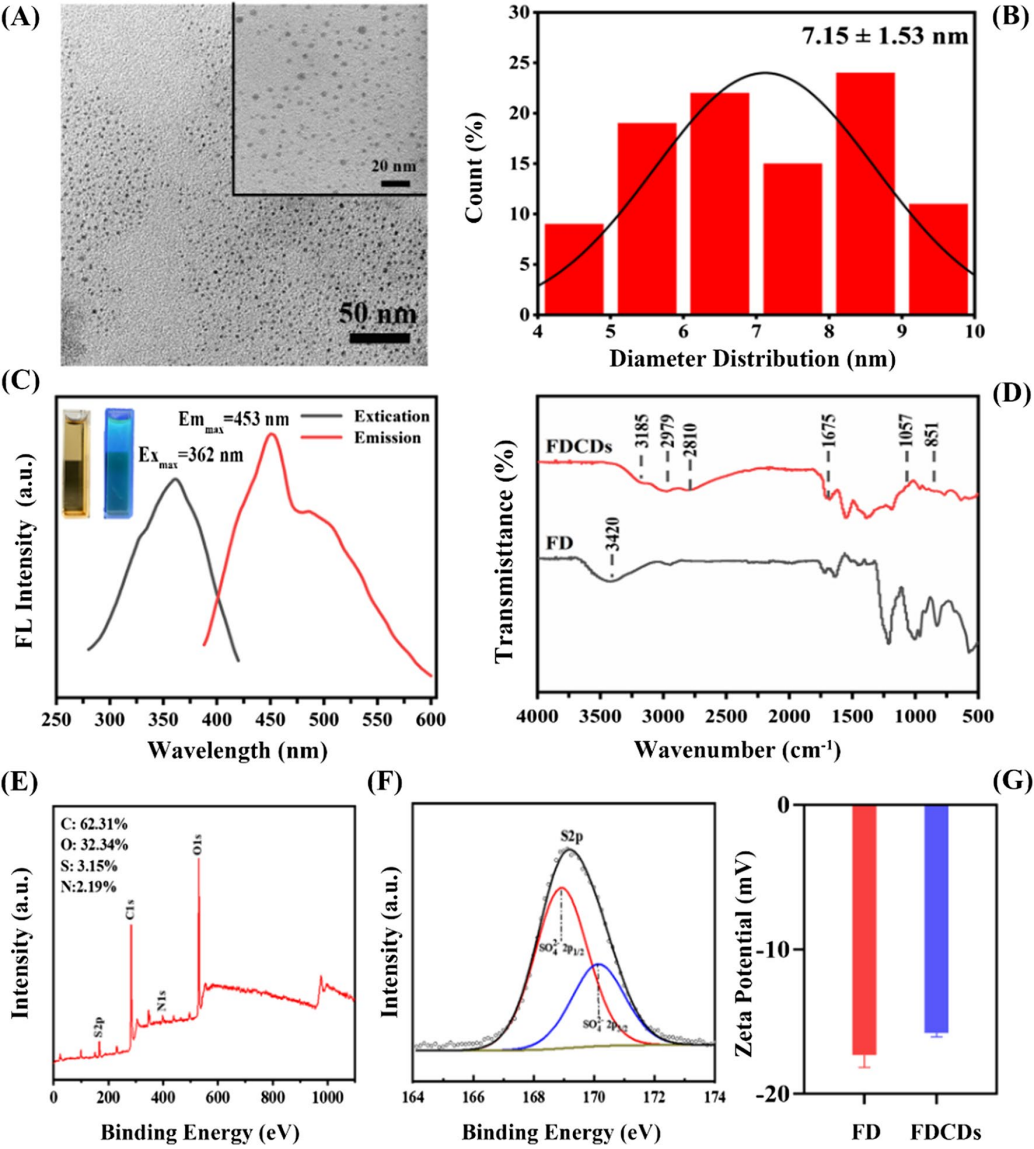
**A** TEM images and **B** particle size distribution of FDCDs. **C** UV–VIS absorption spectra of FDCDs. The inset photos show FDCDs solution under ambient light (left) and 365 nm UV radiation (right), respectively. **D** FT-IR spectra, **E**, **F** XPS survey spectrums, and **G** zeta potentials of FD and FDCDs in DI water

In this work, using FD as a precursor, the dark*-*brown CDs powder was prepared by the hydrothermal method. The shape and size of FDCDs were detected by TEM. As shown in Fig. 1A, the FDCDs exhibited the nanoscale size, dispersed homogeneously, and spherical shape. In Fig. 1B, the diameter distribution of FDCDs was ranging from 4−10 nm, with an average size of 7.15 ± 1.53 nm. This result revealed that FDCDs were synthesized successfully. Further, the FDCDs solution exhibited light brown in ambient light but appeared green-colored luminescence under 365 nm UV light excitation (inset of Fig. 1C). In addition, the excitation and emission fluorescence spectra of the FDCDs were recorded in Fig. 1C. A fluorescence maximum excitation wavelength was 362 nm, and the maximum emission wavelength was 453 nm. This phenomenon confirmed that FDCDs possessed unique optical properties, which was attributed to the large energy discrepancy. The functional groups of the FDCDs were characterized by FT-IR spectroscopy (Fig. 1D). The broad peak between 3600 and 3000 cm^−1^ indicates the stretching vibrations of the O–H group [40]. The peak at 3185 cm^−1^ in the spectrum of FDCDs was related to the O-H bond. The peak at 2979 and 2810 cm^−1^ were attributed to C–H bonds. The peak at 1675 cm^−1^ was associated with the COO− groups. Importantly, the peaks around 1057 and 832 cm^−1^ belonged to the C=S and S=O bonds as well as C–O–S bending vibration, which are associated with the typical biofunctional groups of FD precursor [41].

XPS was used to analyze the surface elemental compositions of FDCDs [42]. As shown in Fig. 1E**/**F, it was found that the contents of C, O, and N were 62.31, 32.34, and 2.19%, respectively. To further confirm the SO_4_^2−^ of FDCDs, S was measured, and the content of S was 3.15%. Moreover, there were two peaks at 168.92 and 170.14 eV, which are both attributed to SO_4_^2−^ bonds.

The surface charges were detected by measuring zeta potential [43]. As shown in Fig. 1G, it was found that the FD and FDCDs were negatively charged and both average zeta potentials were − 17.33 ± 0.69 and − 15.80 ± 0.22 mV, respectively. The negative charge on the surface of FDCDs was attributed to the presence of sulfur groups. Taken together, these results indicate that FDCDs were successfully prepared using FD, which remained the representative SO_4_^2−^ of FD. The size distribution and dispersion stability of small particles were commonly measured by DLS. PDI is one of the quality indicators of a material in terms of particle size distribution. The size of FDCDs detected by DLS was 5.12 nm and the PDI value of them was 0.231 ± 0.014, which both indicate that the size distribution is narrow and monodisperse particles were detected (Additional file 1: Fig. S1, Table S1) [38].

### Anti-bacterial/biofilm activity of FDCDs

**Fig. 2:**
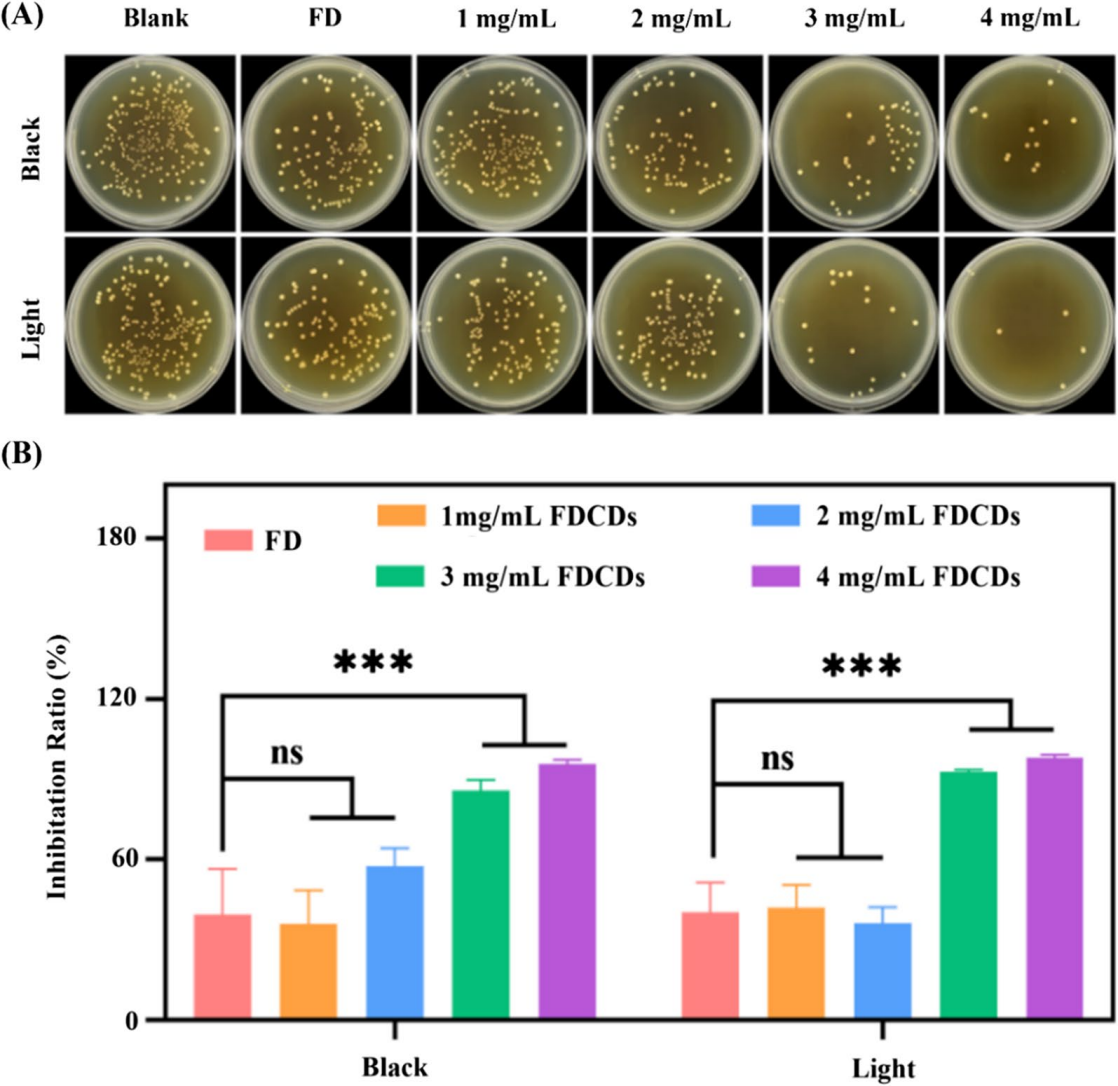
**A** Photographs of survived bacterial colonies of *E. faecalis* treated with precursor FD and different concentrations of FDCDs in dark and light conditions. **B** The inhibition ratios of *E. faecalis* at corresponding concentrations of FD and FDCDs. Data are mean ± SD (n = 3) (ns means no significance, ***p < 0.001)

To investigate the antibacterial activity of FDCDs, the CFU count test was employed. As shown in Fig. 2A, the number of *E. faecalis* colonies on agar plates was not significantly different under visible light illumination and dark conditions. After co-culturing 12 h, the antibacterial differences among the negative control, FD, 1, and 2 mg/mL FDCDs groups were negligible under the same condition. Importantly, the *E. faecalis* colonies on the plates gradually decreased with the increase of FDCD concentration. In Fig. 2B, the antibacterial efficiency in the presence of FD and FDCDs at concentrations of 1–4 mg/mL was calculated. The FD at a concentration of 4 mg/mL showed no significant antibacterial activity. The antibacterial activity of FDCDs with low concentrations (i.e., 1 and 2 mg/mL) was not statistically different from that of the blank control group. The inhibition ratio of the FDCDs concentrations (i.e., 3 and 4 mg/mL) on *E. faecalis* was more than 85%, which was significantly greater than other groups (*p* < 0.001). Compared with precursor FD, FDCDs showed stronger antibacterial properties at the same concentration. In addition, there was no significant difference in the antibacterial efficiency of FDCDs between visible light irradiation and dark conditions. The results indicate that the antibacterial efficiency of FDCDs was closely related to their concentrations.

**Fig. 3:**
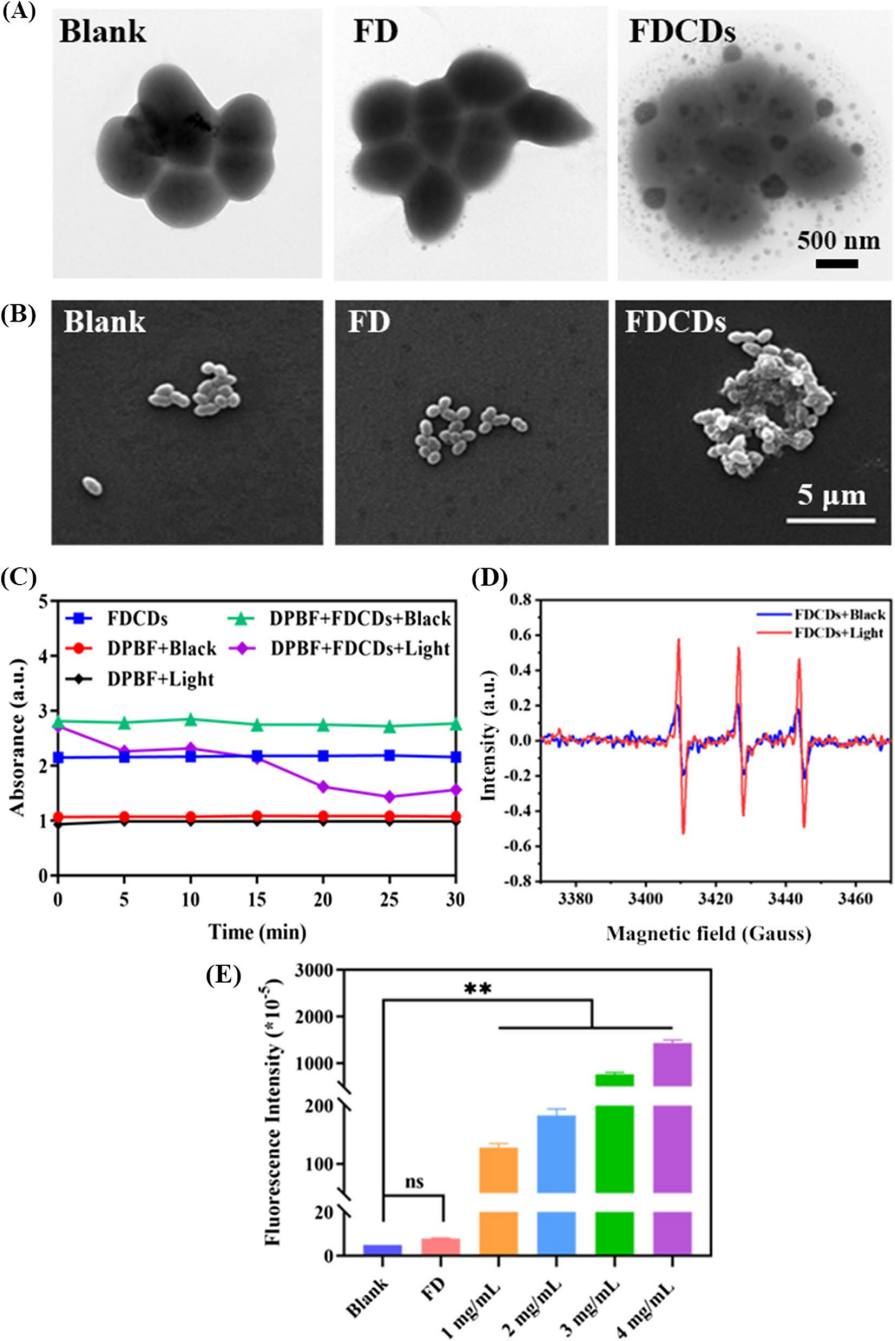
**A** TEM and **B** SEM images of bacterial morphology treated with FD and FDCDs. **C** Extracellular ROS formation induced by FDCDs under light and dark conditions. The absorbance curve of DPBF at 410 nm after different samples were processed for different time points. **D** The production of ^1^O_2_ detected by ESR. **E** Intracellular ROS generation of *E. faecalis* after being treated with FD and FDCDs for 12 h. (*ns* no significance, **p < 0.01)

The integrity of the bacterial wall is an important barrier to ensure their survival [44, 45]. As reported, CDs can increase bacterial permeability and even destroy their wall owing to their extremely small size, the doping of heteroatoms, and specific functional groups [43, 46, 47]. The effect of FDCDs on the morphology of *E. faecalis* was detected using TEM. As depicted in Fig. 3A, the bacteria in the blank group showed a typical spherical-shaped morphology with intact cell walls, and the FD group had no difference from the blank group. Particularly, the permeability of bacteria treated with FDCDs at 3 mg/mL was significantly reduced, which indicates the internal structure of bacteria was damaged. In addition, SEM was also used to observe the morphology of *E. faecalis*. The cell walls of *E. faecalis* in the blank group and FD group were smooth with intact morphology and clear structure. Interestingly, after FDCDs treatment, the bacterial surfaces disintegrated with irregular boundaries (Fig. 3B). Hence, these results suggest that the cell leakage which was caused by the destruction of bacterial walls may be one of the possible antibacterial mechanisms of FDCDs.

In addition, the quenching of DPBF fluorescence was employed to detect the generation of extracellular ROS [48]. As shown in Fig. 3C, the absorbance of DPBF with or without illumination and FDCDs alone was nearly unchanged. The absorbance at 410 nm decreased significantly when DPBF was mixed with FDCDs under visible light, especially within 5 min, which proved the generation of ROS. Conversely, the mixed solution remained unchanged under dark conditions within 30 min. These results imply that FDCDs catalyzed the production of ROS under visible light. Moreover, the intensity of characteristic resonance peaks for ^1^O_2_ increased significantly after irradiation with 365 nm UV light compared with dark conditions (Fig. 3D), which further demonstrates the formation of ^1^O_2_.

Further, DCFH-DA as a ROS assay kit was used to verify whether the FDCDs were able to induce ROS inside bacterial cells. As indicated in Fig. 3E, negligible fluorescence intensity was detected in the blank and FD groups. After incubating with FDCDs for 12 h, the fluorescence intensity in the bacterial cells increased greatly compared to the blank group, and the fluorescence intensity significantly increased with increasing FDCDs concentrations. Therefore, the underlying mechanisms of antibacterial activity of developed nanoparticles were that FDCDs entered bacteria and induced the formation of extracellular/intracellular ROS, which led to the increase of oxidative stress and bacterial permeability, destroyed the bacterial walls, and caused leakage of intracellular fluids [23].

**Fig. 4:**
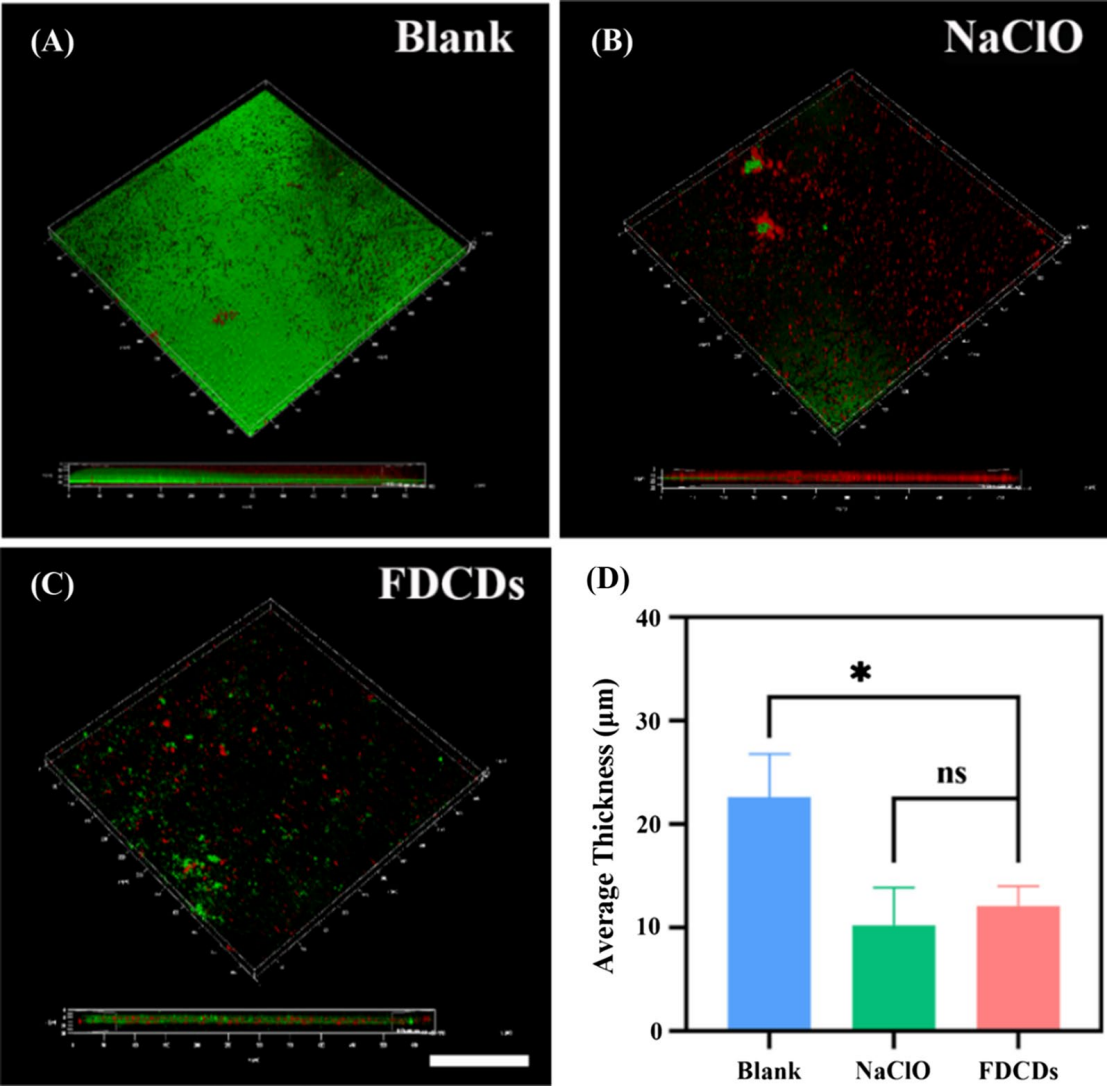
CLSM images of *E. faecalis* biofilms grown for 48 h and treated with samples for 12 h. Biofilms grown in **A** BHI, **B** 1% NaClO, **C** 3 mg/mL FDCDs. Green is live cells. Red is dead cells. Scale bars = 50 μm. **D** The average thickness of *E. faecalis* biofilms. Data are mean ± SD (n = 3) (ns means no significance, *p < 0.05, **p < 0.01)

Biofilm which was composed of microorganisms and extracellular polymeric substance (EPS) can induce persistent bacterial infections [11, 49]. Therefore, it is necessary to explore the effect of FDCDs on *E. faecalis* biofilm. The above experiments have proved that the FDCDs at 3 and 4 mg/mL possessed similar good antibacterial properties. Since their inhibition ratios had no significant difference, a lower concentration (3 mg/mL FDCDs) was chosen for subsequent antibacterial experiments. To investigate how FDCDs affect bacterial biofilms, *E. faecalis* biofilms which were cultured for 48 h were treated by FDCDs at a concentration of 3 mg/mL for 12 h. *E. faecalis* biofilms were stained by a live/dead fluorescence kit and observed their destructive effects by CLSM. As shown in Fig. 4A–C, CLSM images revealed that the entire surface of the glass coverslips was covered with *E. faecalis* biofilm in the blank group. Importantly, the surface coverage and thickness of *E. faecalis* biofilms were significantly decreased when treated with 3 mg/mL FDCDs, which confirms that the bacteria were killed, and related biofilms were destroyed. As shown in Fig. 4D, as compared to the control group, the average thickness of biofilm treated with 3 mg/mL FDCDs decreased significantly. It shows that the damage of 3 mg/mL FDCDs to the biofilm was comparable to clinic used NaClO.

**Fig. 5:**
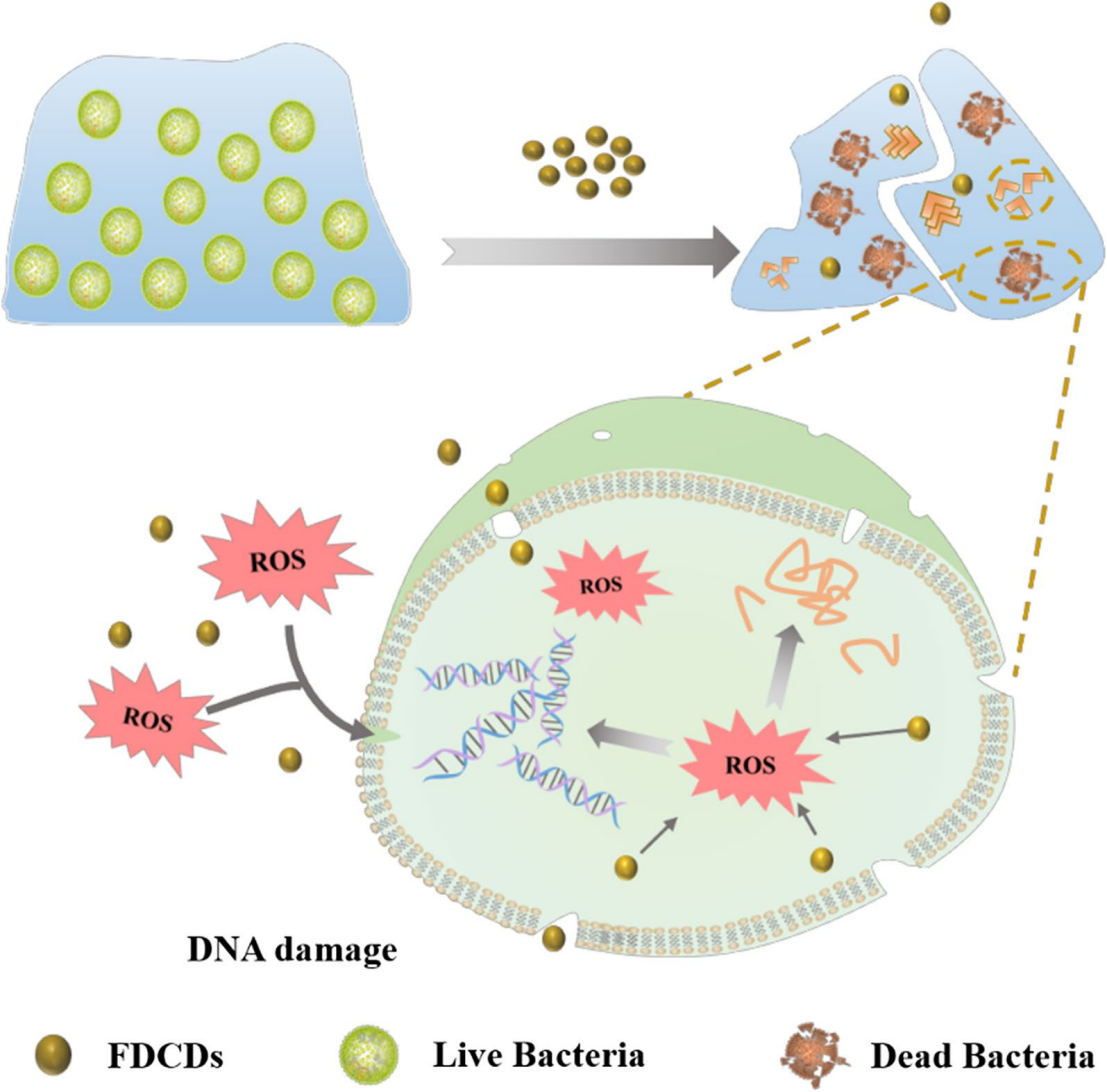
Schematic diagram of antibacterial mechanism of FDCDs

The schematic diagram of the antibacterial mechanism of FDCDs was illustrated in Fig. 5. The raw materials or precursor reagents with antibacterial groups play a critical role in the antibacterial property of developed CDs. Generally, the prepared CDs using the hydrothermal approach could retain the functional groups such as the quinone group of henna, the quaternary ammonium group of glycine betaine, etc. [50–54] It has been demonstrated that sulfated polysaccharide exhibited antibacterial activity, which was related to the sulfate groups [33, 55]. In our case, the extremely small FDCDs with SO_4_^2−^ enhanced the antibacterial and antibiofilm activity compared with FD. As a zero-dimensional nanomaterial, small-sized FDCDs easily stick to the bacterial surface, penetrate the plasma membrane, and lead to damages to intracellular biomolecules [43, 47, 56–58]. Moreover, Liu, et al. revealed that the CDs could generate ^1^O_2_, whereas the intrinsically antibacterial activity of CDs is also vital [59]. In our study, negatively charged FDCDs induced the formation of extracellular/intracellular ROS, which lead to the increase of oxidative stress to destroy intracellular biomolecules and then killed bacteria [60–63]. The antibiofilm results imply that extremely small FDCDs could penetrate in biofilms through their channels, increasing the damage of biofilms structure [11].

### Anti-bacterial/biofilm activity of FDCDs in dentin blocks

Antimicrobial drugs and materials are difficult to penetrate the complex root canal system to remove biofilm efficiently, therefore, the penetration depth in the dentinal tubules is one of the important characteristics of root canal disinfectants [26, 64, 65].

**Fig. 6:**
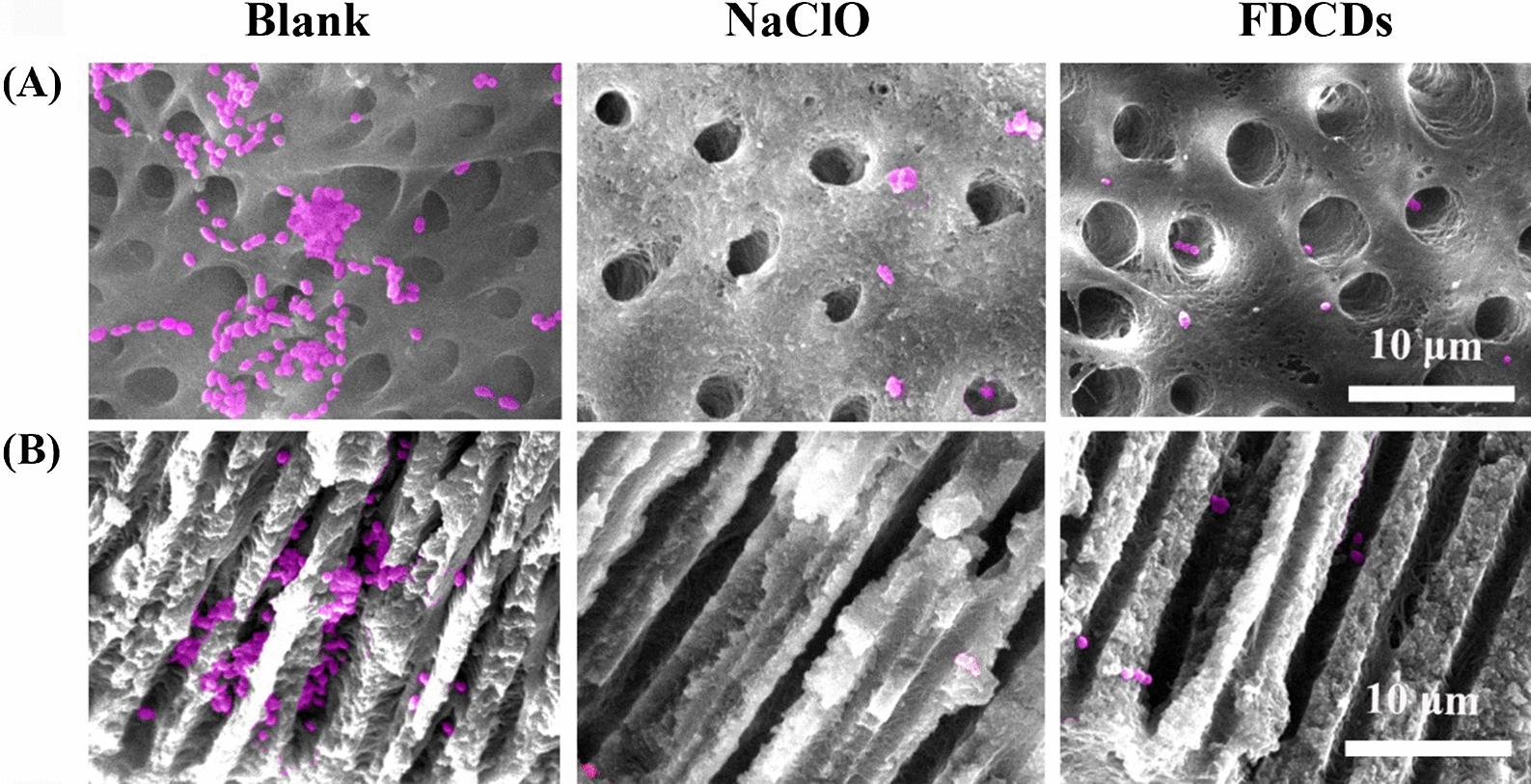
False colored SEM images of *E. faecalis* (pink) on **A** the root canal surface and in **B** the dentinal tubules treated with FDCDs and NaClO

As shown in Additional file 1: Fig. S2, the teeth infiltrated in the FDCDs group displayed brown color, while only the root canals were stained when the FDCDs were injected into the root canals. Although this color was irreversible, it could indicate that the FDCDs were easily adhered to the teeth and penetrate into the dentinal tubules [66]. As shown in Fig. 6 A**/**B, SEM was used to observe the effect of different treatments on *E. faecalis* in the root canal surface and dentinal tubules. Compared to the blank control, FDCDs significantly removed *E. faecalis* on the root canals and in the dentinal tubules, which is comparable to the clinic used NaClO. The root canal surface after the treatment of NaClO became rougher due to its corrosive effect. Therefore, FDCDs efficiently eradicated pathogenic bacteria but had no side effect on the structure of teeth.

### Cytocompatibility and macrophage response of FDCDs

**Fig. 7:**
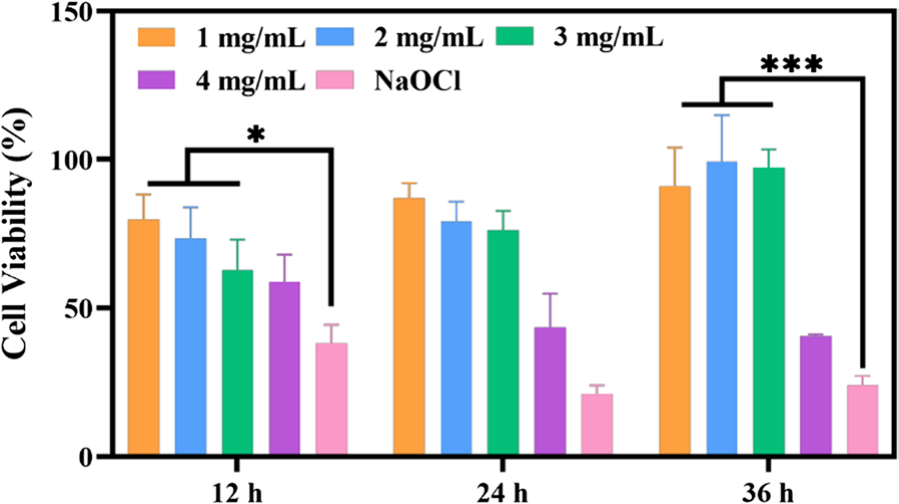
The viability of MC3T3-E1 cells after incubated with FDCDs. Data are mean ± SD (n = 3) (*ns* no significance, *p < 0.05, ***p < 0.001)

Assessment of cytocompatibility is essential to determine whether a nanoparticle is suitable for biomedicine [11, 67–70]. After being treated with FDCDs for 12, 24, and 36 h, the CCK-8 assay was used to investigate the impact of FDCDs on MC3T3-E1 cells viability. As shown in Fig. 7, after the incubation for 12 h, the cell viability was decreased with the increasing concentration of FDCDs from 1 to 3 mg/mL, but the cells remained highly viable (> 50%) [71]. When co-cultured for 24 and 36 h, the cell viability in the concentration up to 3 mg/mL was more than 80%. However, when the concentration of FDCDs reached 4 mg/mL, the cell viability was significantly decreased (< 50%). Therefore, their cell viability effect was increased in a dose-dependent manner. In addition, the cell viability in NaClO was extremely low, indicating its apparent toxicity compared to the FDCDs.

**Fig. 8:**
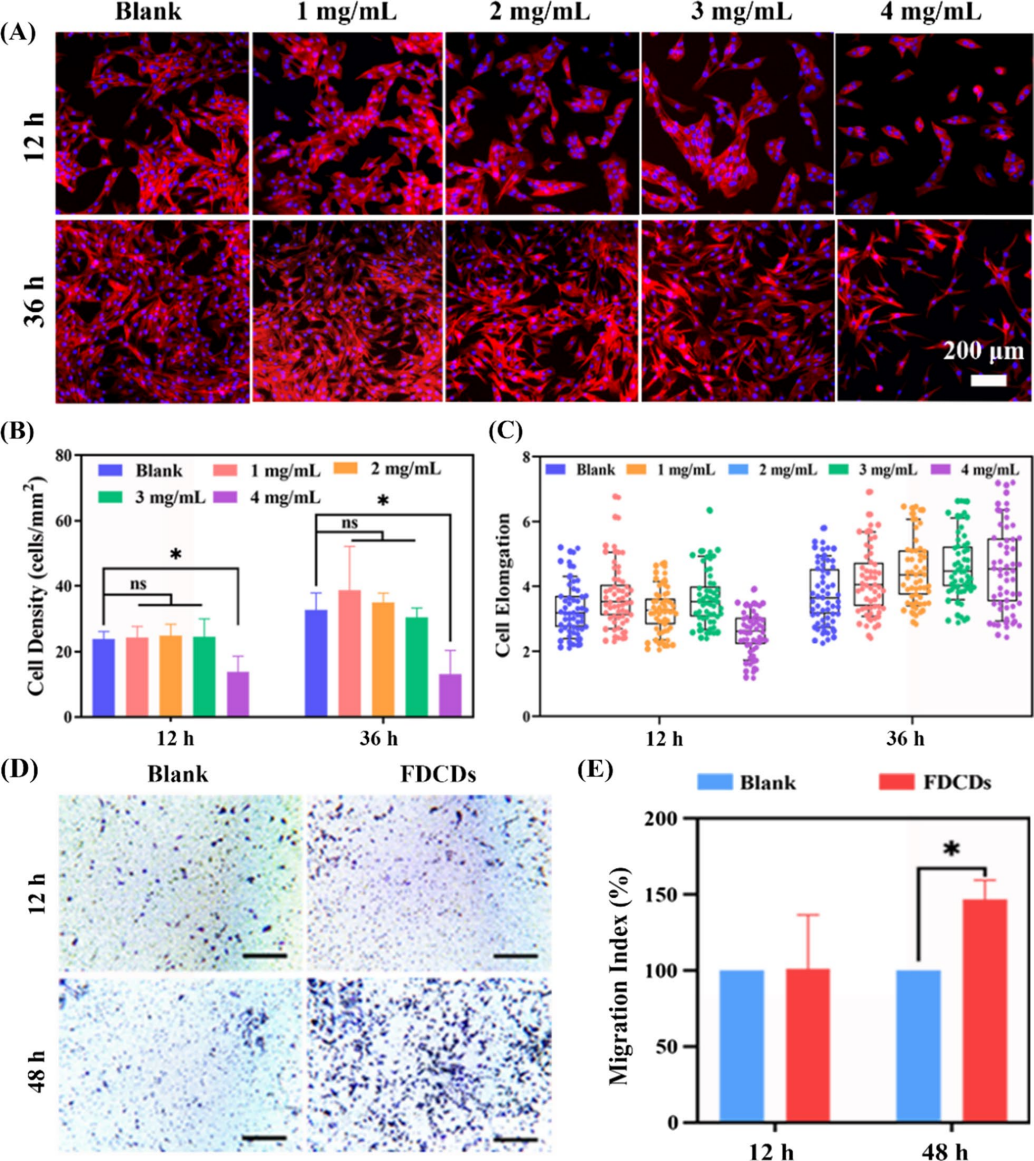
**A** Fluorescent images of MC3T3-E1 cells treated with different concentrations of FDCDs for 12 and 36 h. Red is actin filaments stained by TRITC–phalloidin staining, and blue is the nucleus visualized by DAPI. **B** The density and **C** elongation of MC3T3-E1 cells after co-cultured with FDCDs for 12 and 36 h. **D** Bright-field images of migrated RAW 264.7 cells incubated without or with 3 mg/mL FDCDs (scale bar, 200 μm). **E** Chemotaxis index of RAW 264.7 cells. Data are mean ± SD (n = 3) (*ns* no significance, *p < 0.05, ***p < 0.001)

Furthermore, nanomaterial-induced cell morphology changes represent a critical role in determining cell viability, proliferation, migration, and differentiation [72–74]. After being treated with FDCDs for 12 and 36 h, the actin cytoskeleton and nucleus were stained by fluorescence staining to evaluate MC3T3-E1 cells adhesion and morphology. In Fig. 8A, the cells treated with FDCDs concentrations from 1 to 3 mg/mL had similar typical spindle-like morphology to the blank group. It also shows that the number of attached cells increased significantly, and the shape of cells had no obvious difference with the increase of time from 12 to 36 h. The morphology of cells treated with 4 mg/mL FDCDs changed significantly at 12 h, whereas the cells returned to spindle cells at 36 h. Quantification displays that the cell density in 1 to 3 mg/mL FDCDs was similar to the blank group, while the cell density in 4 mg/mL FDCDs was decreased greatly (Fig. 8B). Figure 8C showed that the elongation in 4 mg/mL FDCDs for 36 h was longer than that in 4 mg/mL FDCDs for 12 h. In addition, there was no significant difference in the elongation among the other experimental groups and the blank group. These results indicate that FDCDs exhibit good cytocompatibility, possessing great potential in the treatment of PEIs.

Macrophages respond rapidly after exposure to nanomaterial, and as the first line of defense against bacterial infection, macrophages induced to migrate by the nanomaterial are able to eliminate bacteria [75–77]. Therefore, the effect of FDCDs on macrophage recruitment deserves attention [78, 79]. As shown in Fig. 8D, the RAW 264.7 cells at the bottom of the polycarbonate membrane co-cultured with FDCDs for 12 and 48 h were stained by the crystal violet staining to detect their migration. Compared with the blank group, there are no significant differences in the number of migrated cells treated with FDCDs for 12 h. However, it was found that FDCDs treatment for 48 h resulted in a significant increase in macrophage migration. The quantitative data in Fig. 8E also supported the above results. FDCDs could affect the migration of macrophages, therefore, it preliminarily inferred that a large number of macrophages could phagocytose bacteria cooperating with the FDCDs to fight bacteria.

## Conclusions

In summary, FDCDs with SO_4_^2^^−^ were successfully prepared by a facile and green hydrothermal strategy. The negatively charged FDCDs possessed superior antibacterial and anti-biofilm activities by generating ROS and altering bacterial permeability. Meanwhile, the FDCDs showed good cytocompatibility and recruited macrophages. According to the isolated tooth experiment, the FDCDs permeated into dentinal tubules and inhibited the *E. faecalis* biofilm. Thus, the designed FDCDs offered a novel strategy to eradicate bacteria, displaying great potential for the therapy of PEIs.

## Supplementary Information

**Additional file 1: Table S1.** Average values of the FDCDs dispersibility index (PDI) in DI water. **Figure S1.** The size distribution of FDCDs detected by DLS. **Figure S2.** The color of isolated teeth cultured with FDCDs for 12h.

## Data Availability

Without restrictions.
