# Supplementary material for: Fucoidan-derived carbon dots against Enterococcus faecalis biofilm and infected dentinal tubules for the treatment of persistent endodontic infections
Source: J Nanobiotechnology. 2022 Jul 14;20:321. doi: 10.1186/s12951-022-01501-x (PMC9281061; doi:10.1186/s12951-022-01501-x)
Supplement: Supplementary file 1 — Additional file 1: Table S1. Average values of the FDCDs dispersibility index (PDI) in DI water. Figure S1. The size distribution of FDCDs detected by DLS. Figure S2. The color of isolated teeth cultured with FDCDs for 12h. [file 12951_2022_1501_MOESM1_ESM.docx]

Supporting information

**Fucoidan-derived carbon dots against Enterococcus faecalis biofilm and infected dentinal tubules for the treatment of persistent endodontic infections**

Shang Tang ^a,b^, Hui Zhang ^a,b^, Li Mei ^b,c^, Yuying Jiang ^b^, Shuai Wang ^a,b^, Mohamed Sayed Hasanin ^e^, Jing Deng *^a,b^, Qihui Zhou *^a,b,c,f^

**Author Affiliations:**

^a^ Department of Stomatology, The Affiliated Hospital of Qingdao University, Qingdao 266003, China.

^b^ School of Stomatology, Qingdao University, Qingdao 266003, China.

^c^ Institute for Translational Medicine, The Affiliated Hospital of Qingdao University, Qingdao University, Qingdao 266021, China.

^e^ Cellulose and Paper Department, National Research Centre, Dokki, 12622, Cairo, Egypt.

^f^ University of Health and Rehabilitation Sciences, Qingdao 266071, China.

*Corresponding author:

Jing Deng, M.M., Professor of Stomatology, Email: dengjing3333@qdu.edu.cn.

Qihui Zhou, Ph.D., Professor of Biomaterials, Phone: +86-17660670299, Email: qihuizhou@qdu.edu.cn.

**Supporting Information**


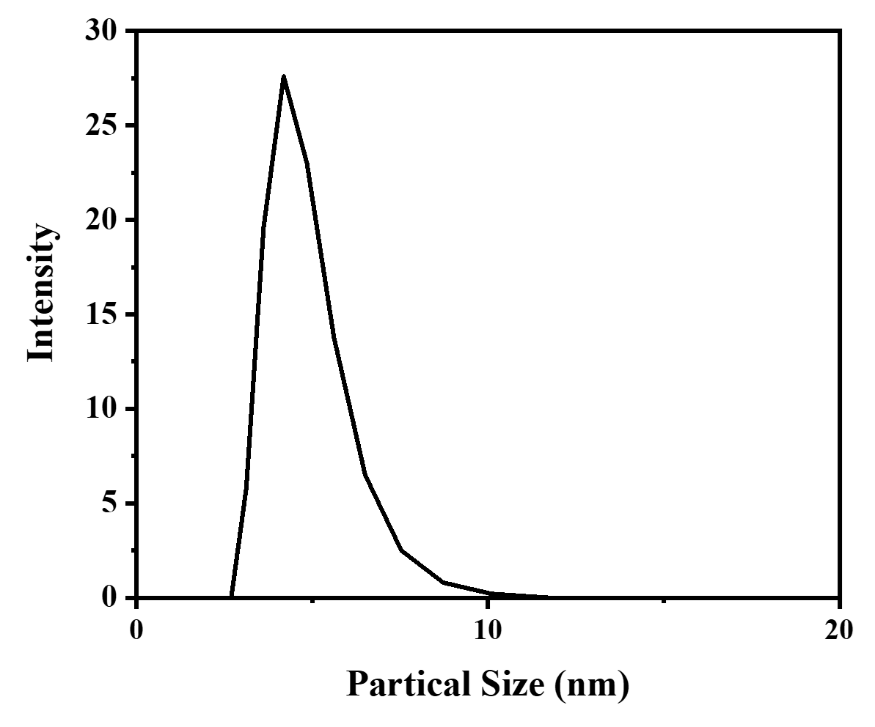


**Figure S1.** The size distribution of FDCDs detected by DLS.

**Table S1.** Average values of the FDCDs dispersibility index (PDI) in DI water.

| Sample | polydispersity index  (PDI) ^1^ | | | Average value | Standard deviation (SD) |
| --- | --- | --- | --- | --- | --- |
| FDCDs | 0.222 | 0.219 | 0.251 | 0.231 | 0.014 |

^1^ PDI value of three replicates.


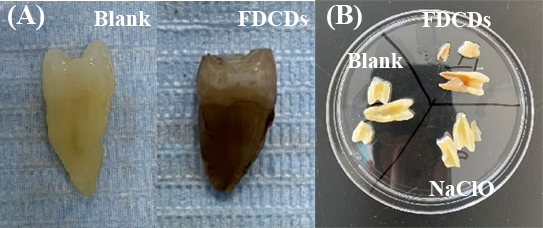


**Figure S2**. (A) The color of isolated teeth after 12 h of immersion in FDCDs solution. (B) The color of isolated teeth 12 h after FDCDs solution was injected into the root canal.
